# Vesiculobullous eruption with loncastuximab tesirine in a patient with relapsed follicular lymphoma

**DOI:** 10.1016/j.jdcr.2024.08.013

**Published:** 2024-09-02

**Authors:** Riyad N.H. Seervai, Craig Y. Okada, Stephanie J. Mengden-Koon, Noah I. Hornick

**Affiliations:** a Department of Dermatology, Oregon Health & Science University, Portland, Oregon; b Department of Hematology and Medical Oncology, Oregon Health & Science University, Portland, Oregon

**Keywords:** dermatologic toxicity, follicular lymphoma, interface dermatitis, loncastuximab tesirine, oncologic dermatology, vesiculobullous dermatitis

## Introduction

Loncastuximab tesirine (LT) is an antibody-drug conjugate (ADC) that has shown efficacy in patients with relapsed/refractory B-cell malignancies.^1^^,^^2^ It comprises an anti-cluster of differentiation (CD)19 antibody linked to the pyrrolobenzodiazepine dimer SG3199, a potent inter-strand minor-groove DNA crosslinking agent, and has prolonged cytotoxic activity against CD19^+^ cells.^3^ Cutaneous toxicities to LT are limited due to its specificity and short half-life, with reactions including a nonspecific maculopapular or pustular rash, photosensitivity, and ulcers.^4^ Two cases of a photodistributed eruption and subsequent vesicular lesions and edema have been reported with LT.^5^ Here, we report a third case of blistering lesions associated with LT, with similar clinical and histopathological findings, in a patient with relapsed follicular lymphoma. Our case expands on the initial series in corroborating salient features of this eruption and in reporting the results of therapeutic intervention and a timeline to resolution.

## Case presentation

The patient is a 55-year-old man with a history of follicular lymphoma first diagnosed in 2014. He was started on rituximab with 2 years of maintenance therapy and experienced a good partial response. He developed progressive lymphadenopathy in late 2019 that progressed despite additional rituximab and trials of 8 cycles of obinutuzumab (anti-CD20), 6 cycles of bendamustine, and a 5-month trial of the EZH2 inhibitor tazemetostat.^6^ Repeat lymph node biopsy showed loss of CD20 expression. At this point, he was enrolled in the LOTIS-7 clinical trial of the anti-CD19 antibody loncastuximab teserine with the anti-CD79 B antibody polatuzumab vedotin,^7^ with his first treatment in March 2023.

**Fig 1:**
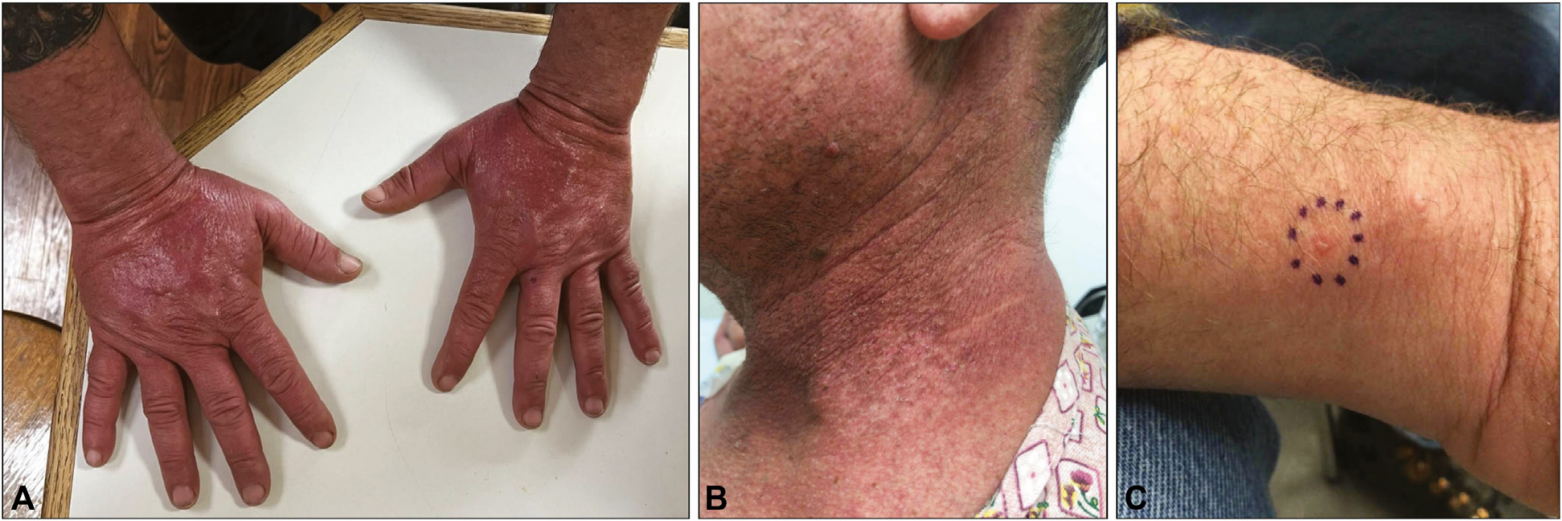
Initial presentation of the patient’s lesions. **A,** Diffuse erythema and swelling of bilateral hands. **B,***Red*, poikilodermatous plaques with dusky scale in a photodistribution on the neck. **C,** Tense vesicle on the right ulnar wrist. Histopathology findings of the biopsied lesion are shown in Fig 2.

**Fig 2:**
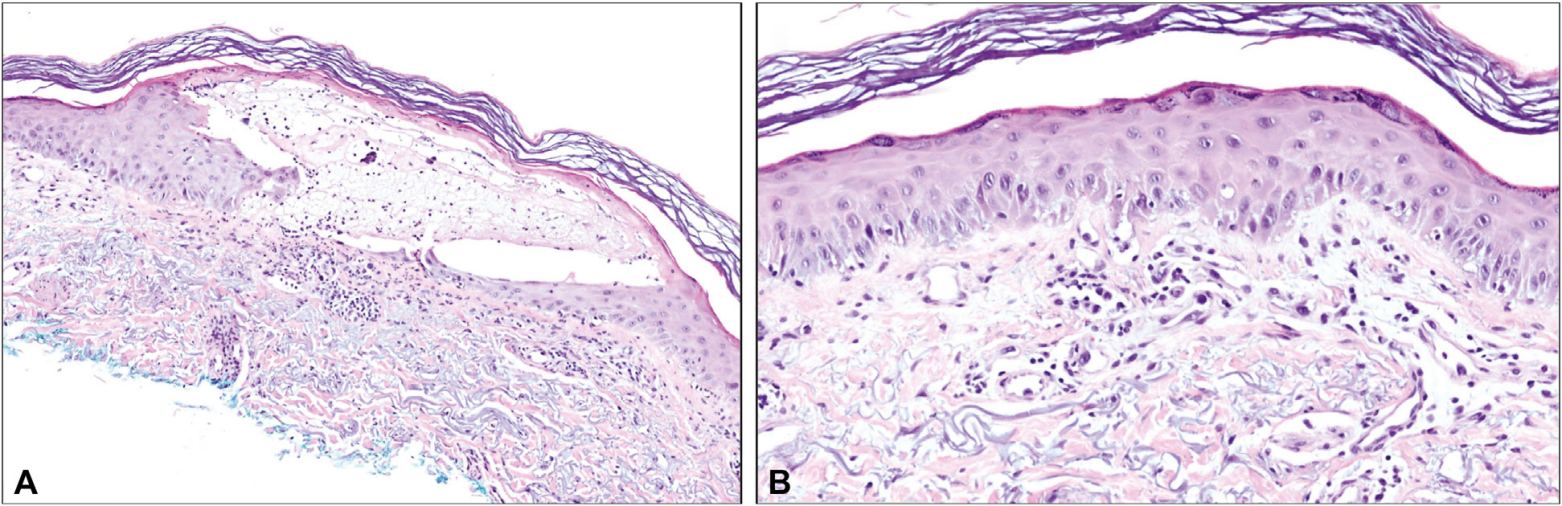
Histopathology of the vesicle on the right ulnar wrist. **A,** Intraepidermal vesiculation with necrosis and exocytosis of lymphocytes, histiocytes, neutrophils, and eosinophils. Hematoxylin and eosin, original magnification ×40. **B,** Interface dermatitis with basal vacuolization and dyskeratosis, lymphohistiocytic infiltrate, and basket-weave orthokeratosis. Hematoxylin and eosin, original magnification ×40.

During the third cycle of treatment, the patient developed a nonpruritic rash on the head and neck that progressed to include redness and swelling of both hands (Fig 1, *A*) and the feeling of a “sunburn.” A week later, he reported worsening with a “burning” sensation, mild pruritus, swollen hands, and sloughing of the skin. He was referred to our clinic, where physical exam showed confluent red, slightly poikilodermatous, thin plaques with dusky/gray exfoliative scale in a photo-distributed pattern and a solitary, 1 millimeter tense vesicle on the right ulnar wrist (Fig 1, *B* and *C*). He reported regular sunscreen application and worked at night without significant sun exposure. Biopsy of the wrist lesion showed intraepidermal vesiculation and interface vacuolar dermatitis in the context of a mixed lymphohistiocytic infiltrate (Fig 2), with the direct immunofluorescence finding of granular C3 and rare dot-like IgA deposition along the basement membrane zone. PAS and herpes simplex virus/varicella zoster virus immunostains were negative. Given these findings, a vesiculobullous reaction to loncastuximab was favored over other considerations: herpes simplex virus/varicella zoster virus and dermatophytes were not detected; histology and immunofluorescence were not characteristic of bullous pemphigoid, porphyria cutanea tarda, or connective tissue disease (in particular, intraepidermal vesiculation is uncharacteristic of connective tissue diseases). The clinical appearance was considered less typical of dyshidrotic dermatitis or allergic contact dermatitis. Twice daily clobetasol 0.05% ointment to the hands and continued strict sun protection were recommended.

**Fig 3:**
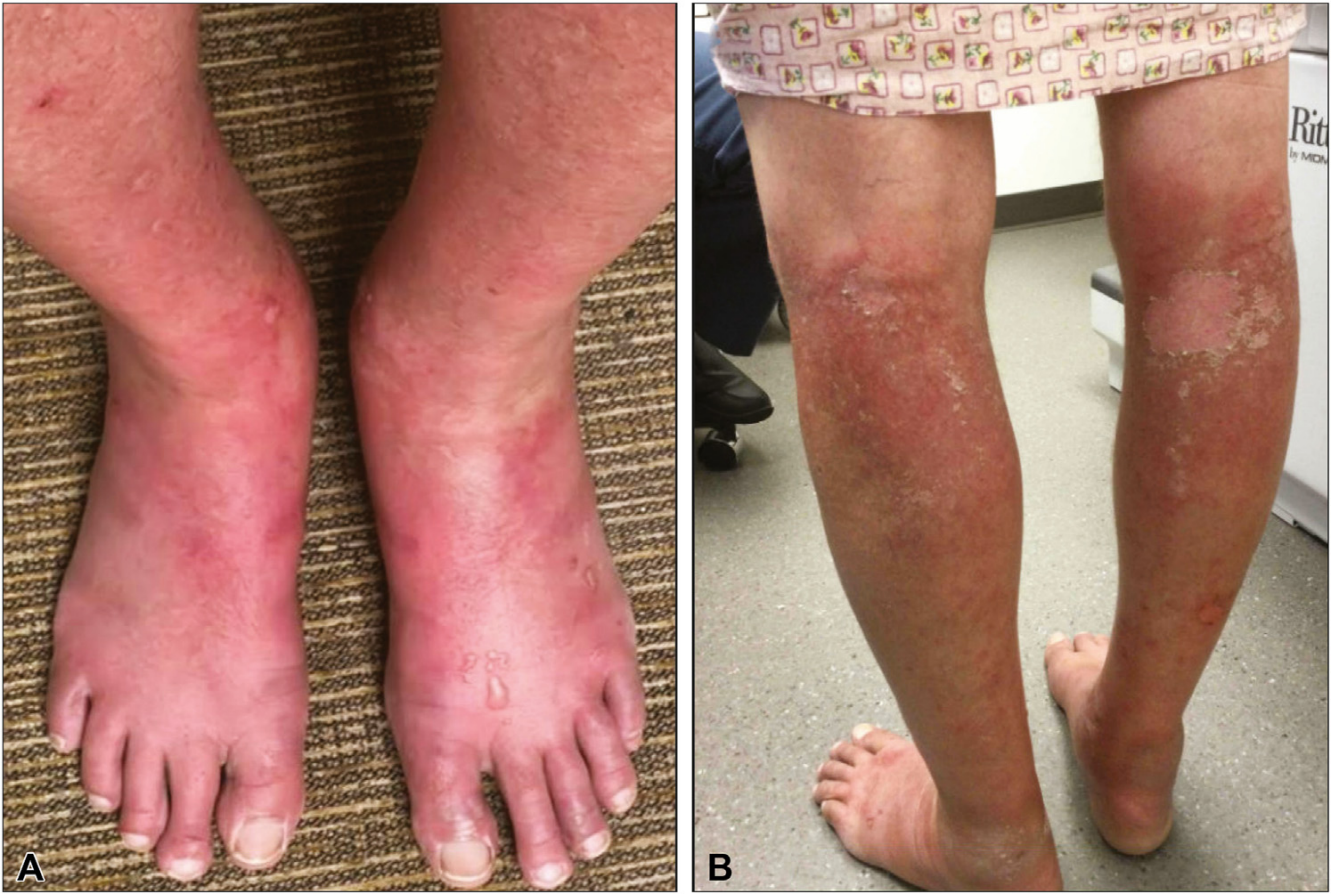
Progression of vesiculobullous eruption. **A,** Diffuse erythema with semi-tense vesicles on the dorsal feet and ankles. **B,** Dusky *purple* plaques with scale in the bilateral popliteal fossae, with erosions on the right calf from minimal trauma.

At follow-up 6 weeks later (4 weeks after his last infusion), his rash had spread beyond his head/neck and upper extremities, and he reported lower extremity edema, blisters, and a painful/burning sensation with occasional itch. Exam showed numerous semi-tense vesicles on a bright pink to red base on the dorsal feet and ankles, dusky firm edematous plaques with epidermal peeling in bilateral popliteal fossae, and superficial abrasion/ulceration on the right calf from minimal trauma (Fig 3). The loncastuximab and polatuzumab were held, and he was removed from the clinical trial due to progression of the skin lesions and worsening transaminase elevation. He was started on a 1-week prednisone taper starting at 0.5 mg/kg (40 mg) daily. Within 4 days of prednisone initiation, his rash and edema had improved, and they were completely resolved at 3-month follow-up.

## Discussion

Vesiculobullous reactions are exceedingly rare with monoclonal antibody therapy for hematologic malignancies, with isolated cases of bullous dermatitis and epidermolysis bullosa acquisita reported with rituximab.^4^ Here, we report a third case of a blistering/vesicular eruption with unique clinical and histopathologic findings in a B-cell lymphoma patient receiving LT (Table I). Pruritus, photosensitivity, morbilliform or papulopustular eruptions, and lower extremity edema have previously been reported with LT.^4^^,^^8^ Bullous lesions with vesicular/interface dermatitis, absence of dermal edema, and focal/granular deposition of IgA along the basement membrane have not been reported except in one previous report.^5^ As previously noted, the vesicular lesions had a unique presentation and pathology distinguishing them from bullous eruptions associated with peripheral edema and toxic erythema of chemotherapy. Both patients in the previous report received LT monotherapy, while our patient also received polatuzumab vedotin. However, cutaneous reactions with this agent are rare and overlap with other ADCs containing monomethyl auristatin e as their cytotoxic payload, and bullous eruptions with this agent have not been reported. Although bullous reactions to the Nectin-4-targeted ADC enfortumab vedotin (which also contains monomethyl auristatin e) have been reported, these are thought to be secondary to the targeting of Nectin-4, which is involved in cell-cell adhesion in the epidermis.^4^

**Table I:** Patient demographic characteristics, oncologic history, dermatological clinical and pathologic features, management, and clinical course

	Case 1^5^	Case 2^5^	Our case
Age/gender	98/F	68/M	55/M
Diagnosis	MZL → DLBCL	FL → DLBCL	Relapsed FL
Previous treatment	R-CHOP, BR, ibrutinib	R-CHOP, R-GemOx, radiation	Rituximab, obinutuzumab, bendamustine, tazemetostat
Treatment at time of reaction	LT monotherapy	LT monotherapy	LT and polatuzumab vedotin
Time to onset of vesicular rash	3 cycles (7 weeks)	4 cycles (12 weeks)	3 cycles (8 weeks)
Other skin manifestations	Preceding photodistributed rash involving other sites; new-onset bilateral lower extremity edema	Preceding photodistributed rash involving other sites; worsening of existing bilateral lower extremity edema	Preceding photodistributed nonpruritic rash on head/neck spreading caudally; new-onset lower extremity edema
Biopsy H&E	Subepidermal vesicular dermatitis, no dermal edema	Subepidermal vesicular dermatitis, interface dermatitis, no dermal edema	Vesiculobullous dermatitis, interface dermatitis, no dermal edema
Biopsy DIF	Fine granular IgA at basement membrane	Fine grains of IgA alone basement membrane zone	Focal dot-like/granular IgA along basement membrane zone
Management	Supportive	Supportive	Topical clobetasol, prednisone 0.5 mg/kg × 1 wk
Treatment status/outcome	Discontinued with resolution of symptoms, no recurrence	Discontinued, patient deceased	Discontinued with improvement in symptoms, no recurrence

*BR*, Bendamustine and rituximab; *DIF*, direct immunofluorescence; *DLBCL*, diffuse large B-cell lymphoma; *F*, female; *FL*, follicular lymphoma; *H&E*, hematoxylin and eosin; *LT*, loncastuzimab tesirine; *M*, male; *MZL*, marginal zone lymphoma; *R-CHOP*, rituximab, cyclophosphamide, hydroxydaunorubicin (doxorubicin), oncovin (vincristine), and prednisolone; *R-GemOx*, rituximab, gemcitabine, and oxaliplatin.

All patients in the previous report received supportive treatment, and LT was discontinued, although not specifically due to the cutaneous eruption. Our patient’s rash was one of two concomitant toxicities contributing to the discontinuation of LT, the other being transaminase elevations to 5 times the upper limit of normal. His eruption did improve with topical clobetasol and prednisone, although his LT had been discontinued by the time of initiation of prednisone, and it is unclear which of these was responsible for his improvement.

In summary, this case contributes to the very limited body of literature on the cutaneous toxicities associated with LT and provides other dermatologists with the results of our therapeutic intervention. Reported adverse reactions to other tesirine-containing ADCs include morbilliform/acneiform eruptions, pruritus, and photosensitivity with camidanlumab tesirine (anti-CD25)^9^ and rovalpituzumab tesirine (anti-delta-like protein 3).^10^ Isolated cases of dose-limited “bullous dermatitis” have been reported with rovalpituzumab tesirine,^10^ although the reactions were not described. Further study is needed to elucidate the mechanism of this and similar eruptions.

## Conflicts of interest

Dr Hornick reports a relationship with Shook Hardy & Bacon LLP that includes consulting or advisory. Drs Seervai, Okada, and Mengden-Koon have no conflicts of interest to declare.
